# Lipoma arborescens in a 22-year-old male: A case report

**DOI:** 10.1097/MD.0000000000040801

**Published:** 2024-12-06

**Authors:** Mingyang Li, Ling Ding, Qilong Nie, Zeping Jiang

**Affiliations:** a The Eighth Clinical Medical College, Guangzhou University of Chinese Medicine, Foshan, Guangdong, China; b Foshan Hospital of Traditional Chinese Medicine, Guangzhou University of Chinese Medicine, Foshan, Guangdong, China.

**Keywords:** arthroscopic surgery, case report, knee joint lesion, lipoma arborescens, MRI diagnosis, synovial hyperplasia

## Abstract

**Rationale:**

Lipoma arborescens (LA) is a rare, benign proliferative disorder of the synovial lining that typically affects middle-aged individuals, predominantly in the knee joint. However, its occurrence in younger patients is unusual and may pose unique diagnostic and therapeutic challenges. This case report aims to highlight the clinical, imaging, and therapeutic aspects of LA in a young adult, thereby expanding the understanding of its presentation in this age group.

**Patient concerns:**

A 22-year-old male presented with acute swelling and pain in the right knee following a minor sprain. Although the symptoms persisted and worsened over several days, the patient was particularly concerned about his prognosis and the possibility of recurrence. Given the rarity of LA, he expressed anxiety about being diagnosed with such an uncommon condition at his age and feared the potential impact on his future mobility. He was also apprehensive about the risks of surgery and questioned why he, at such a young age, was affected by this rare condition.

**Diagnoses:**

Initial clinical assessment was inconclusive, leading to further investigations. Magnetic resonance imaging findings suggested a meniscal tear and the presence of LA, characterized by fat globules and joint effusion, which was confirmed during arthroscopic surgery.

**Interventions:**

The patient underwent arthroscopic surgery involving trimming of the medial meniscus and debridement of the synovium. Surgical findings included extensive synovial proliferation and intra-articular fat globules, which were histologically confirmed as LA.

**Outcomes:**

Postoperative recovery was successful, with significant improvement in joint function and alleviation of symptoms. Follow-up magnetic resonance imaging confirmed the absence of abnormal synovial proliferation.

**Lessons:**

This case report underscores the diagnostic and therapeutic challenges associated with LA in a young patient, highlighting the importance of considering LA in differential diagnoses of acute knee swelling, especially when initial evaluations are inconclusive. Through a combination of imaging, arthroscopic intervention, and histopathological confirmation, this case illustrates an effective approach for accurately diagnosing and managing LA. By documenting this atypical presentation in a young adult, this report contributes to the limited literature on LA in younger populations, aiming to enhance clinical awareness and guide future cases involving similar joint lesions.

## 1. Introduction

Lipoma arborescens (LA), also known as synovial lipomatous villous proliferation, is a relatively rare clinical condition. It results from various factors causing chronic reactive hyperplasia of the joint synovium and sub-synovial adipose tissue. LA is considered a tumor-like lesion rather than a true neoplasm.^[1]^ The primary manifestations of LA include progressive joint swelling, intermittent swelling, and reduced skin sensation.^[2,3]^ This condition can occur at any age, but it is most common in middle-aged and elderly individuals. Additionally, LA is considered to affect men more frequently than women.^[4,5]^ It typically involves larger joints, with the knee being the most commonly affected.^[6]^ Currently, there are few reports on the diagnosis and treatment of this condition using arthroscopy both domestically and internationally. At the Department of Orthopedics of Foshan Traditional Chinese Medicine Hospital, one case of knee joint LA was treated with arthroscopic debridement in addition to pharmacotherapy, achieving favorable clinical outcomes.

## 2. Case presentation

### 2.1. Patient background and presenting complaints

A 22-year-old male patient was admitted to Foshan Traditional Chinese Medicine Hospital on March 10, 2024, due to swelling and pain in the right knee joint with limited mobility, persisting for 4 days following a sprain. The patient reported that the symptoms began after he suddenly twisted his right knee during a fitness exercise, leading to pain and restricted movement. Initially, he sought medical attention at another hospital, where X-rays showed no fractures, although the specific treatment was not documented. The swelling progressively worsened, prompting further consultation at our hospital.

### 2.2. Physical examination and initial imaging findings

**Figure 1.**
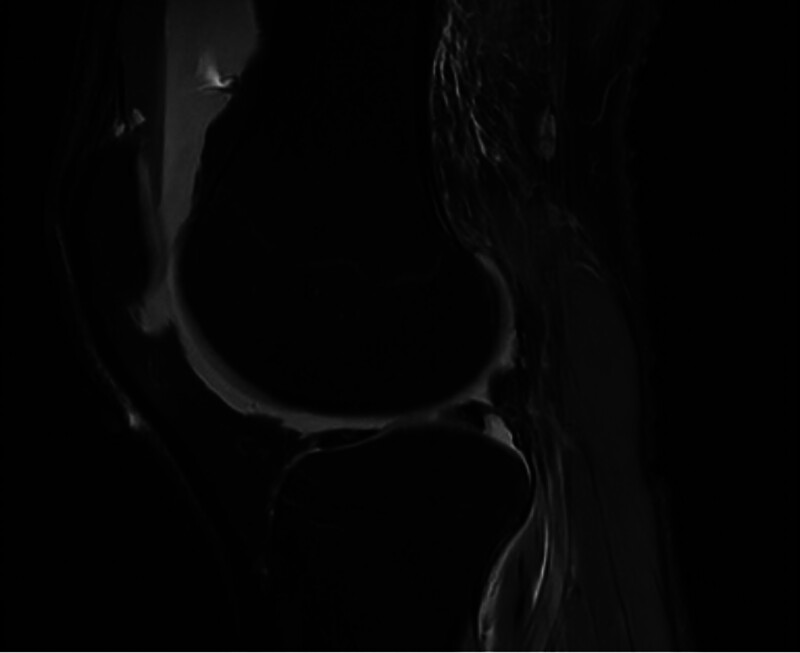
Sagittal view: there was also significant joint effusion and lipohydarthrosis with fat globules, suggesting lipoma arborescens.

**Figure 2.**
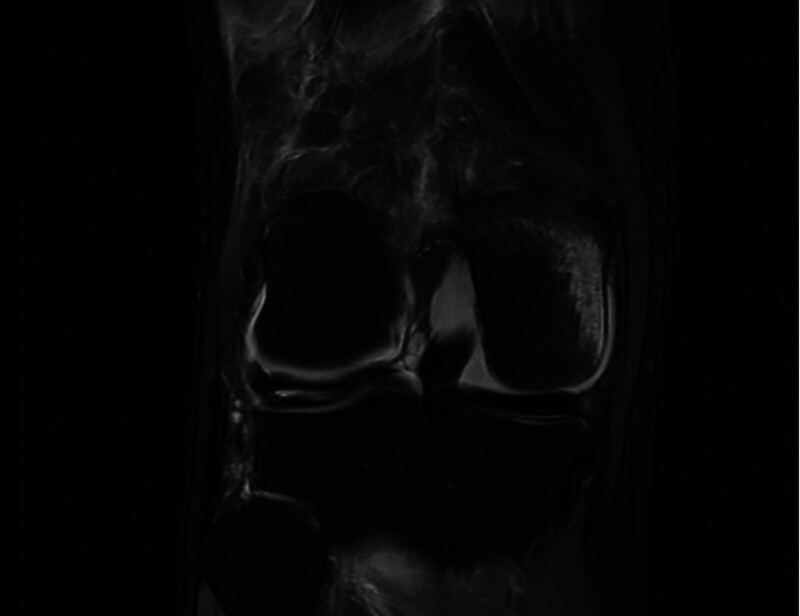
Coronal view: T1-weighted coronal images showing large fat-signal mass in the rear aspect of the distal femur.

Upon admission, physical examination revealed equal leg lengths, swelling, redness, and warmth in the right knee, with mild tenderness upon palpation. The patellar tap test was positive, while the drawer test and varus and valgus stress tests were negative. Toe blood supply and sensation were normal. MRI revealed a tear from the anterior horn to the posterior horn of the lateral meniscus of the right knee, along with a meniscal cyst. There was also significant joint effusion and lipohydarthrosis with fat globules, suggesting LA (Figs. 1 and 2). After excluding surgical contraindications, the patient underwent arthroscopic trimming and suturing of the medial meniscus of the right knee, along with exploratory and debridement surgery of the right knee joint.

### 2.3. Surgical intervention and pathological findings

**Figure 3.**
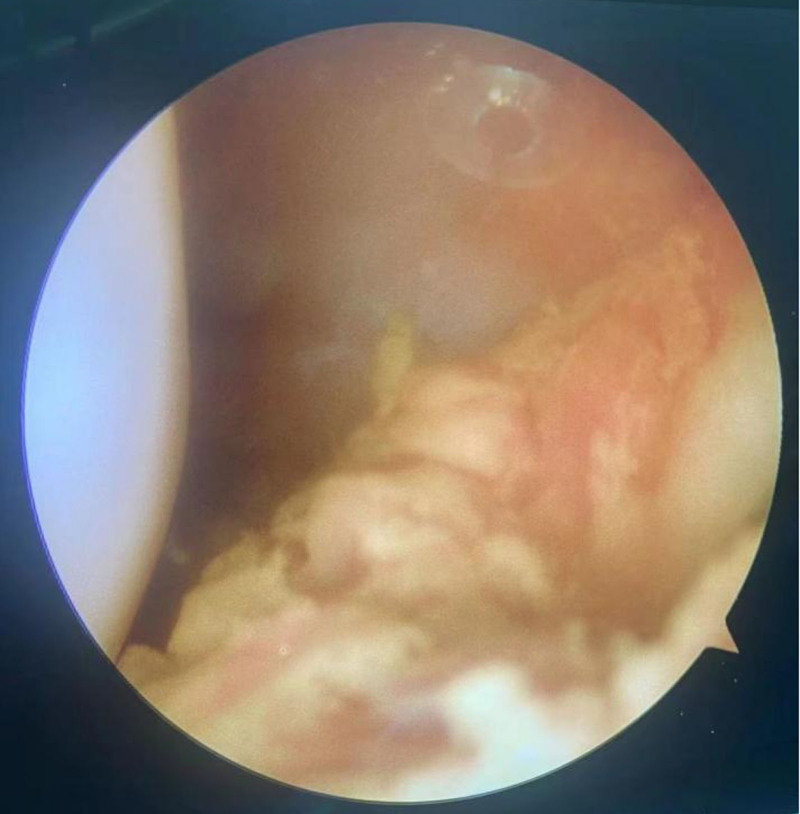
Arthroscopic findings a significant number of intra-articular fat globules.

The surgery was performed under caudal epidural anesthesia combined with intravenous anesthesia, using an anteromedial and anterolateral approach to the right knee. Two 8 mm parapatellar incisions were made on either side of the patellar tendon, and an arthroscope was inserted. The suprapatellar bursa, medial gutter, medial joint space, intercondylar notch, lateral joint space, lateral gutter, and suprapatellar bursa were sequentially examined. Arthroscopic findings included approximately 80 mL of hemarthrosis, extensive synovial proliferation, fatty attachments, and a significant number of intra-articular fat globules (Fig. 3). The cruciate ligaments appeared intact, and there was no noticeable damage to the medial meniscus. However, a horizontal tear extending from the anterior horn to the posterior horn of the lateral meniscus, along with meniscal degeneration, was observed. Arthroscopic procedures included lateral meniscus reshaping and debridement of the proliferative synovium within the joint. Synovial tissue samples were collected and sent for pathological examination.

**Figure 4.**
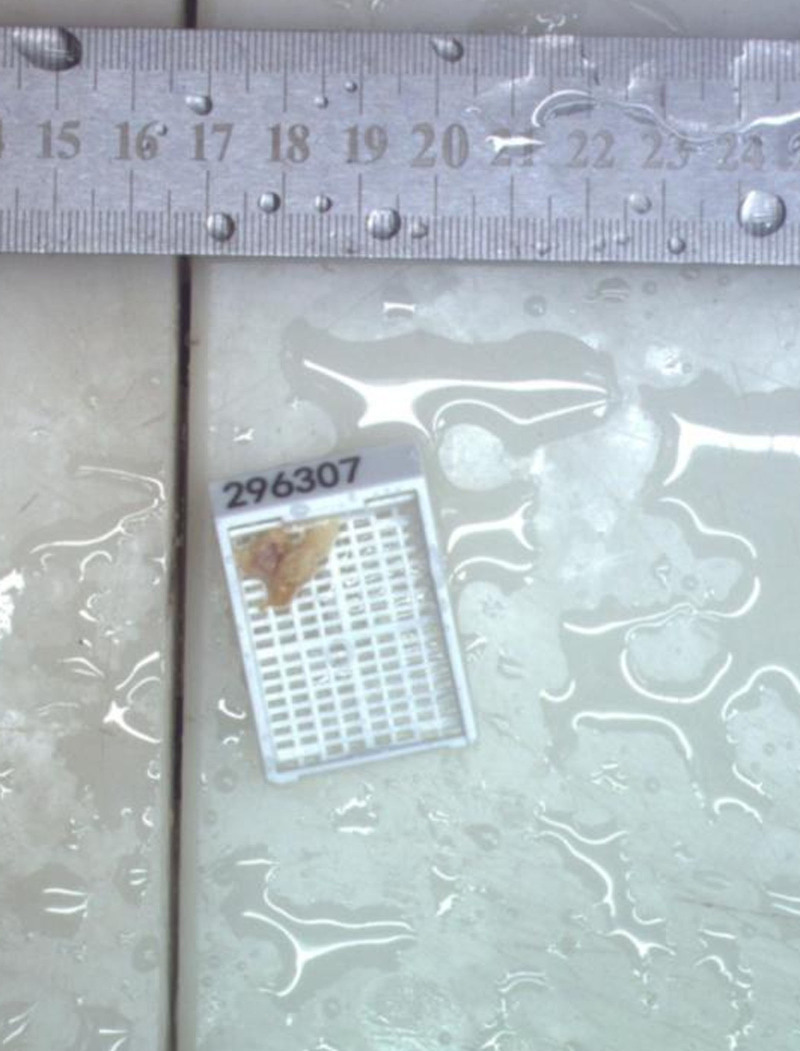
Pathological specimen: the specimen consisted of gray-yellow tissue measuring 1 cm × 0.8 cm × 4.4 cm.

Pathological examination: Gross examination: The specimen consisted of gray-yellow tissue measuring 1 cm × 0.8 cm × 4.4 cm (Fig. 4). Microscopic findings: Examination revealed extensive synovial hyperplasia with fatty attachments, numerous intra-articular fat globules, and meniscal degeneration.

**Figure 5.**
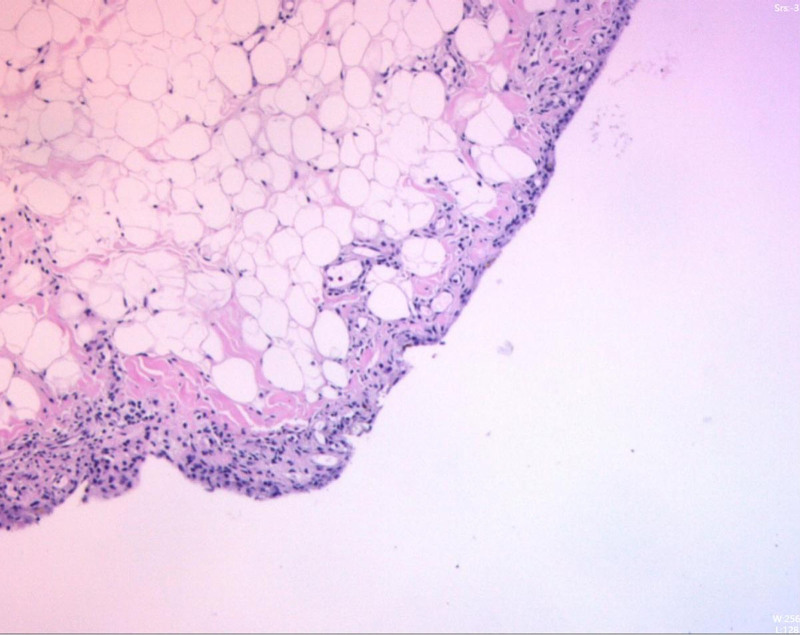
Pathological examination: chronic synovitis with lipoma arborescens-like proliferation.

**Figure 6.**
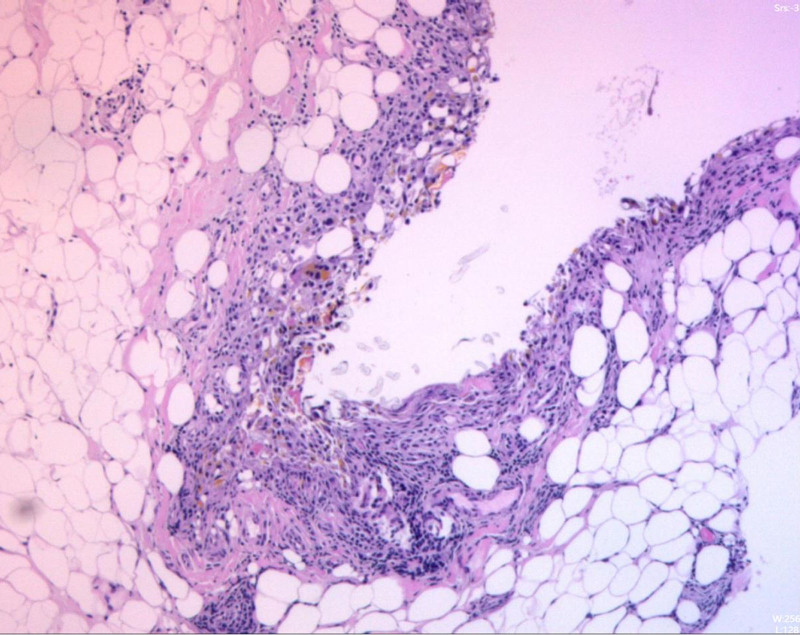
Pathological examination: chronic synovitis with lipoma arborescens-like proliferation.

**Figure 7.**
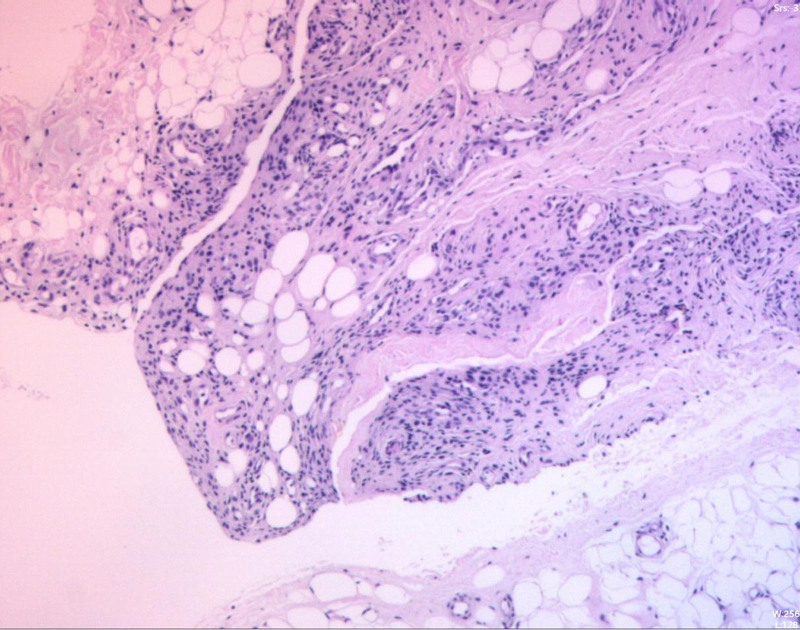
Pathological examination: chronic synovitis with lipoma arborescens-like proliferation.

Pathological diagnosis: Chronic synovitis with lipoma arborescens-like proliferation. Polarized light microscopy did not show any crystals (Figs. 5–7).

### 2.4. Management and follow-up

The patient was managed with arthroscopic surgery to address the structural abnormalities observed in imaging studies. The procedure included trimming and suturing of the medial meniscus, as well as extensive synovial debridement to remove proliferative tissue. During surgery, marked synovial proliferation and numerous intra-articular fat globules were observed, aligning with the diagnosis of LA. Synovial tissue samples were collected for histopathological confirmation.

Postoperative care emphasized a comprehensive rehabilitation and wound management plan to optimize recovery and prevent complications. The plan included:

Early mobilization: Starting within 24 hours postsurgery, the patient began passive range-of-motion exercises to prevent joint stiffness and adhesions, aiming to maintain joint flexibility and minimize scar tissue formation.

Gradual weight-bearing: Over the first 2 weeks, the patient was gradually introduced to partial weight-bearing exercises, progressing to full weight-bearing as tolerated. This step-wise approach allowed the knee joint to adapt to load while minimizing the risk of reinjury.

Strengthening exercises: From the third week postsurgery, targeted quadriceps and hamstring strengthening exercises were incorporated to support knee stability and function, emphasizing muscle balance around the knee to reduce stress on the joint.

Wound care and infection monitoring: Special attention was directed towards wound healing. Regular dressing changes were performed, and the wound area was kept clean and dry to prevent infection. The patient and healthcare team closely monitored for signs of wound discharge, redness, or swelling indicative of infection. Routine checks of serum infection markers, such as C-reactive protein and white blood cell count, were conducted to detect any early signs of systemic infection, enabling prompt intervention if necessary.

Functional training: By the end of the first month, functional exercises were added to restore balance, proprioception, and coordinated movement, preparing the patient to safely resume regular physical activities.

On June 20, 2024, a telephone follow-up was conducted to assess the patient’s recovery status. The patient reported significant improvement in knee joint function with complete resolution of symptoms, including swelling and pain. An MRI performed at his local hospital confirmed the absence of synovial proliferation or joint effusion, with no signs of LA recurrence. The surgical wound had healed without complications, and the patient’s mobility and knee stability were fully restored, enabling him to safely return to unrestricted daily activities.

## 3. Outcomes and follow-up

### 3.1. Case summary

This case highlights the successful diagnosis and management of LA in a young patient, presenting an atypical occurrence of this rare synovial proliferative condition. Following a sudden knee injury, the patient experienced persistent joint swelling and pain, which were ultimately attributed to LA. The diagnosis was confirmed through MRI and arthroscopic examination, revealing characteristic fat globules and synovial proliferation. Arthroscopic trimming and synovial debridement effectively alleviated symptoms, and a structured postoperative rehabilitation program supported optimal functional recovery. At the 3-month follow-up, the patient reported complete symptom resolution and unrestricted knee mobility, with MRI findings confirming no recurrence of LA.

### 3.2. Follow-up plan

Upon discharge, the healthcare team thoroughly discussed the follow-up plan with the patient, ensuring he understood the importance of regular monitoring and imaging to detect any signs of recurrence. The patient expressed that returning to our hospital for follow-up and MRI evaluations may not be feasible due to geographic constraints. However, he committed to following the recommended follow-up schedule at his local hospital, where he would arrange for MRI scans in line with our plan. Our medical team will conduct regular telephone follow-ups to monitor his progress and provide professional guidance as needed.

Short-term follow-up: Every 3 months during the first postoperative year: The patient is advised to undergo physical examinations at his local hospital to assess knee stability, range-of-motion, and any potential signs of synovial swelling or effusion. An MRI scan is recommended at the 6-month mark to verify the absence of synovial proliferation and to monitor for any recurrence. Our medical team will conduct telephone follow-ups at these intervals to review his condition, MRI results, and any symptoms reported, ensuring continuity of care and timely intervention if necessary.

Annual follow-up: From the second postoperative year onward: The patient should have an annual knee joint evaluation, including physical examination and imaging (MRI or ultrasound) if symptoms recur or if any abnormalities are noted on examination. Our team will also reach out annually to assess his knee function and to provide any additional recommendations based on his progress.

Guidance on physical activity:

First 3 months: The patient is advised to avoid high-impact or intense physical activities, focusing instead on gentle, low-impact exercises that support joint mobility and muscle strength.

3 to 6 months: Moderate activities, such as walking, light jogging, or cycling, may be gradually introduced based on tolerance. High-impact sports and heavy lifting should still be avoided.

After 6 months: If MRI confirms stable joint status with no recurrence, the patient may resume regular activities, gradually increasing the intensity under the guidance of his local healthcare provider to ensure knee stability.

This follow-up plan is designed to facilitate consistent monitoring through collaborative care, allowing the patient to safely undergo necessary evaluations locally while remaining connected to our team for professional guidance and support.

## 4. Discussion

The etiology of LA is currently unclear, but it is often clinically associated with trauma and tumors.^[7]^ Diagnosing LA typically requires a thorough assessment of clinical symptoms and auxiliary examinations. Ultrasound can help identify abnormal structures within fatty tissue, such as the boundaries and internal characteristics of the lipoma. MRI, with its high resolution for soft tissues, can provide detailed images of the lipoma’s size, shape, and its relationship with surrounding tissues, aiding in the exclusion of other soft tissue tumors.^[8]^ Pathologically, the fatty tissue in LA may show signs of inflammatory cell infiltration, adipocyte atypia, or fibrosis. Biopsy is useful to exclude malignant tumors or other conditions like panniculitis.^[9]^

Previous reports on LA are very rare. Michio Hamanishi^[10]^ reported a case of a 17-year-old female diagnosed with lipomatous synovitis after experiencing right hip pain following a physical education class. Initially suspected to have a femoral neck stress fracture, subsequent imaging and MRI revealed fatty villous proliferation within the hip joint. The abnormal fatty tissue was successfully excised using the Ganz abduction technique. Pathological examination confirmed that the synovial stroma was expanded by mature adipose tissue in a tree-like or villous configuration, accompanied by chronic inflammation. Hajar Ouazzani Chahdi^[11]^ described a case of a 23-year-old male who presented with persistent swelling and effusion in the left knee. MRI showed fatty villous projections within the synovium, appearing as high signal on T1-weighted images and low signal on PD-weighted images, with no enhancement after fat suppression. There was also diffuse synovial enhancement and moderate joint effusion. Three months later, the patient underwent arthroscopic total synovectomy, and histological examination confirmed the diagnosis of knee joint LA. This case highlights the importance of early diagnosis and treatment based on imaging characteristics, with MRI being the preferred method due to its high sensitivity for detecting adipose tissue abnormalities.

Differential diagnosis: (1) giant cell tumor of the tendon sheath (GCTTS) is a common soft tissue tumor and is considered a benign lesion.^[12]^ This tumor primarily occurs in the tendon sheaths of the hands and feet. Clinically, it presents as a slowly enlarging mass on the fingers or other locations, with a firm texture, clear boundaries, and limited mobility. MRI is the gold standard for diagnosing GCTTS, as it can clearly display the tumor’s size, shape, and whether it invades adjacent tissues. Diagnosis typically requires a biopsy or histopathological examination following surgical excision.^[13,14]^ In general, the treatment for GCTTS is surgical removal. Early diagnosis and complete excision help reduce the recurrence rate. Although benign, patients should undergo regular follow-up to monitor for possible recurrence. (2) Pigmented villonodular synovitis is a rare proliferative joint disease primarily affecting the synovium. It is characterized by abnormal proliferation of synovial cells and the deposition of hemosiderin-laden macrophages. Clinically, it presents with joint swelling, pain, and restricted movement. MRI is crucial for diagnosing pigmented villonodular synovitis, as it can show synovial thickening, nodular lesions, and typical low-signal intensity deposits within the joint. Arthroscopy allows direct visualization of villous synovial proliferation and brownish pigmentation. Tissue biopsy is essential for definitive diagnosis. Due to the potential for recurrence, patients require long-term monitoring and follow-up.^[15,16]^ (3) Synovial hemangioma is a rare benign vascular lesion that primarily occurs in the synovial tissue of joints, tendon sheaths, or bursae. This tumor is composed of blood vessels and may sometimes cause hemarthrosis due to vascular rupture. Synovial hemangioma typically affects children or adolescents, presenting with intermittent joint swelling and pain. MRI is a critical diagnostic tool, as it can display the vascular structures and their relationship with surrounding tissues, showing high-signal flow dynamics on imaging. Arthroscopy can directly observe intra-articular lesions such as vascular proliferation and blood leakage. Tissue biopsy is the gold standard for definitive diagnosis.^[17,18]^

This case report has certain limitations. As a single-case study, its findings may have limited generalizability to broader populations. Additionally, the short-term follow-up period restricts our ability to comment on long-term recurrence rates and overall prognosis, which may require further longitudinal studies. Finally, the diagnosis and treatment in this case heavily relied on advanced imaging and arthroscopic techniques, which may limit the applicability of these findings in settings with limited access to such resources. Recognizing these constraints provides a more balanced perspective on the study’s scope and highlights areas for future investigation.

This case report describes a 22-year-old male who sought medical attention following a knee sprain. MRI initially diagnosed a meniscal tear but did not rule out the possibility of LA. The diagnosis was later confirmed through arthroscopic surgery and pathological examination. This case underscores the critical reliance on imaging techniques and pathological examination for diagnosing unusual joint lesions, providing valuable guidance for clinicians dealing with similarly complex joint diseases. However, given the limited sample size of this study, being a single case, its findings may not broadly represent the clinical characteristics of all patients with knee LA.

## 5. Patient perspective

In a recent telephone follow-up, the patient shared his reflections on the treatment journey. Prior to this episode, he had never experienced knee pain, so when he initially twisted his knee, he assumed it was a minor injury and did not seek immediate, comprehensive care. He received initial treatment at his local hospital, but his symptoms showed no improvement, prompting him to seek further evaluation at our hospital. Upon diagnosis with this rare condition, the patient experienced significant anxiety, marked by insomnia and concern over the severity of his illness. He worried that this condition might severely impact his ability to engage in physical activities or limit his future athletic potential, which was particularly distressing given his young age.

The patient also expressed fear upon learning he would require arthroscopic surgery, as this was his first surgical procedure, and he was apprehensive about the recovery process and whether he could return to his pre-injury level of activity. Through comprehensive discussions with our medical team, he gradually accepted his diagnosis and gained an understanding of the condition’s benign nature and favorable prognosis. Our team provided reassurance regarding his postsurgical recovery, emphasizing that with proper rehabilitation, he could resume his normal level of physical activity.

At the most recent follow-up, the patient reported feeling very satisfied with his recovery. He expressed happiness and confidence in his current condition, noting that he had no discomfort and was optimistic about his ability to return to unrestricted activities.

## Author contributions

**Conceptualization:** Mingyang Li, Ling Ding, Qilong Nie.

**Funding acquisition:** Zeping Jiang.

**Writing – original draft:** Mingyang Li.

**Writing – review & editing:** Zeping Jiang.
